# Supplementary material for: Nickel(II)-catalyzed living polymerization of diazoacetates toward polycarbene homopolymer and polythiophene-block-polycarbene copolymers
Source: Nat Commun. 2022 Feb 10;13:811. doi: 10.1038/s41467-022-28475-6 (PMC8831484; doi:10.1038/s41467-022-28475-6)
Supplement: Supplementary file 1 — Supplementary Information [file 41467_2022_28475_MOESM1_ESM.pdf]

Supplementary Information

Nickel(II)-Catalyzed Living Polymerization of Diazoacetates toward Polycarbene Homopolymer and Polythiophene-*block*-Polycarbene Copolymers

*Li Zhou,[†] Lei Xu,[†] Xue Song, Shu-Ming Kang, Na Liu, and Zong-Quan Wu**

Department of Polymer Science and Engineering, School of Chemistry and Chemical Engineering, and Anhui Key Laboratory of Advanced Catalytic Materials and Reaction Engineering, Hefei University of Technology, Hefei 230009, Anhui Province, China

*E-mail: zqwu@hfut.edu.cn

Supplementary methods ········ S3

Measurements ········ S3

Materials ········ S3

Synthetic procedure ········ S4-S14

AFM, TEM, and DLS measurements ········ S14

DFT computational details ········ S14

Supplementary Table 1. Results for the polymerizations using BT–Ni(dppp)Cl ········ S15

Supplementary Table 2. Energies of the DFT optimized minima and transition states ········ S16

Supplementary Fig. S1-S2. SEC traces of poly-$\mathbf{1a}_{m}$s ········ S17

Supplementary Fig. S3-S10. Characterization data of BT-Ni(dppp)Cl ········ S18-S21

Supplementary Fig. S11. SEC traces of poly-$\mathbf{1a}_{50}$ and poly($\mathbf{1a}_{50}$-*b*-$\mathbf{1a}_{30}$) ········ S22

Supplementary Fig. S12-S13. ^{13}C NMR and FT-IR spectra of poly-$\mathbf{1a}_{50}$ ········ S22-S23

Supplementary Fig. S14. MALDI-TOF mass spectrum of poly-$\mathbf{1a}_{20}$ ········ S23

Supplementary Fig. S15-S23. SEC, ^{1}H NMR, and FT-IR of polycarbenes ········ S24-S28

Supplementary Fig. S24. SEC traces of poly($\mathbf{2}_{20}$-*b*-$\mathbf{1a}_{n}$)s ········ S28

Supplementary Fig. S25. FT-IR spectrum of poly($\mathbf{2}_{20}$-*b*-$\mathbf{1a}_{40}$) ········ S29

Supplementary Fig. S26-S27. SEC of poly-$\mathbf{2}_{m}$, poly($\mathbf{2}_{m}$-*b*-$\mathbf{1a}_{n}$)s, and poly($\mathbf{2}_{m}$-*b*-$\mathbf{1b}_{n}$)s ··· S29-S30

Supplementary Fig. S28-S29. ^{1}H NMR and FT-IR of poly($\mathbf{2}_{20}$-*b*-$\mathbf{1b}_{40}$) ········ S30-S31

Supplementary Fig. S30. Photographs of poly($\mathbf{2}_{20}$-*b*-$\mathbf{1b}_{40}$) under room and UV light ········ S31

Supplementary Fig. S31-S32. TEM images and DLS trace of poly($\mathbf{2}_{20}$-*b*-$\mathbf{1b}_{40}$) ········ S32

Supplementary Fig. S33-S34. Intrinsic reaction coordinate calculation results ········ S33

Supplementary Fig. S35-S37. DFT optimized geometry of the transition states ········ S34-S35

Supplementary Fig. S38-S40. ^{1}H NMR spectra of **1a**, **1b**, and **1d** ········ S35-S36

Supplementary references ········ S37

Supplementary methods

Measurements. NMR spectra were recorded using a Bruker 600 MHz spectrometer {H} spectrometer operated in the Fourier Transform mode. Chemical shifts are reported in delta (δ) units and expressed in parts per million (ppm) downfield from tetramethylsilane (TMS) using the residual solvent proton as an internal standard. Size exclusion chromatography (SEC) was performed on Waters 1515 pump and Waters 2414 differential refractive index (RI) detector (set at 40 °C) using a series of two linear TSK gel GMHHR-H columns. Molecular weight (M_n) and its dispersity (M_w/M_n) data were reported relative to polystyrene standards. The eluent was tetrahydrofuran (THF) at a flow rate of 0.8 mL/min. FT-IR spectra were recorded on Perkin-Elmer Spectrum BX FT-IR system using KBr pellets. Matrix assisted laser desorption ionizations spectroscopy with time-of-flight detection mass spectroscopy (MALDI-TOF MS) measurements were performed on a Bruker Reflex III using dithranol as a matrix and sodium trifluoroacetate as an ion source. UV-vis spectra were performed on a UNIC 4802 UV/VIS double beam spectrophotometer in 1.0 cm length quartz cell. Emission spectra were recorded using a Hitachi F-4600 fluorescence spectrophotometer. Atomic force microscope (AFM) was performed on a Cypher S microscope (Oxford Instruments, Asylum Research). Transmission electron microscopy (TEM) was performed on a JEM-2100F operating at 200 kV accelerating voltage. Dynamic light scattering (DLS) was recorded using a Nano-ZS 90 Zetasizer of Malvern (UK) instrument.

Materials. All solvents were obtained from Sinopharm. Co. Ltd., and were purified by the standard procedures before use. All chemicals were purchased from Aladdin, Sinopharm, and Sigma-Aldrich Chemical Co. Ltd., and were used as received without further purification otherwise denoted. Monomers **1a**, **1b**, and **1d** were prepared according to literatures with modifications.[1] Monomer **1c** was obtained from Adamas Co. Ltd. 2-Bromo-3-hexyl-5-

iodothiophene was purchased from TCI. Co. Ltd. The P3HT homopolymer was prepared according to the literature.[2] All the chemical structures were confirmed by ^{1}H NMR spectra. All the purchased and resynthesized compounds was analytical reagent.

Synthetic procedure

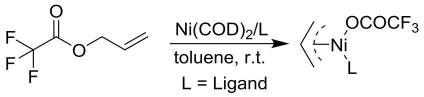

Supplementary equation 1. Synthesis of π-allylnickel(II) catalysts.

The synthetic procedure for π-allyl–Ni(L_1)($OCOCF_3$) catalyst. The catalyst was prepared according to the reported literatures.[3] A flame-dried Schlenk flask charged with π-allyl trifluoroacetate (0.05 mL, 0.36 mmol), bis(1,5-cyclooctadiene)nickel(0) [Ni$(COD)_2$, 0.10 g, 0.36 mmol], and toluene (14.0 mL) was sealed with a rubber septum. The mixture was stirred at room temperature for 20 min under dry N_2 atmosphere. Then, ligand L_1 (Ming-phos, 1.0 M in toluene, 0.37 mL, 0.37 mmol) was introduced *via* a gastight syringe at 0 °C. The mixture was stirred at room temperature for 1 h to afford a solution of π-allyl–Ni(L_1)($OCOCF_3$) catalyst, which was directly used for the polymerizations without further purification.

The synthetic procedure for π-allyl–Ni(L_2)($OCOCF_3$) catalyst. The catalyst was prepared according to the reported literatures.[3] A flame-dried Schlenk flask charged with π-allyl trifluoroacetate (0.05 mL, 0.36 mmol), bis(1,5-cyclooctadiene)nickel(0) [Ni$(COD)_2$, 0.10 g, 0.36 mmol], and toluene (14.0 mL) was sealed with a rubber septum. The mixture was stirred at room temperature for 20 min under dry N_2 atmosphere. Then, ligand L_2 (Xiao-phos, 1.0 M in toluene, 0.37 mL, 0.37 mmol) was introduced *via* a gastight syringe at 0 °C. The mixture was stirred at room temperature for 1 h to afford a solution of π-allyl–Ni(L_1)($OCOCF_3$) catalyst, which was directly used for the polymerizations without further purification.

The synthetic procedure for π-allyl–Ni(L_3)($OCOCF_3$) catalyst. The catalyst was prepared according to the reported literatures.[3] A flame-dried Schlenk flask charged with π-allyl trifluoroacetate (0.05 mL, 0.36 mmol), bis(1,5-cyclooctadiene)nickel(0) [Ni($COD)_2$, 0.10 g, 0.36 mmol], and toluene (14.0 mL) was sealed with a rubber septum. The mixture was stirred at room temperature for 20 min under dry N_2 atmosphere. Then, ligand L_3 (1,4-bis(diphenylphosphino)butane (dppb), 1.0 M in toluene, 0.37 mL, 0.37 mmol) was introduced *via* a gastight syringe at 0 °C. The mixture was stirred at room temperature for 1 h to afford a solution of π-allyl–Ni(L_3)($OCOCF_3$) catalyst, which was directly used for the polymerizations without further purification.

The synthetic procedure for π-allyl–Ni(L_4)($OCOCF_3$) catalyst. The catalyst was prepared according to the reported literatures.[3] A flame-dried Schlenk flask charged with π-allyl trifluoroacetate (0.05 mL, 0.36 mmol), bis(1,5-cyclooctadiene)nickel(0) [Ni($COD)_2$, 0.10 g, 0.36 mmol], and toluene (14.0 mL) was sealed with a rubber septum. The mixture was stirred at room temperature for 20 min under dry N_2 atmosphere. Then, ligand L_4 (1,1'-bis(diphenylphosphion)ferrocene (dppf), 1.0 M in toluene, 0.37 mL, 0.37 mmol) was introduced *via* a gastight syringe at 0 °C. The mixture was stirred at room temperature for 1 h to afford a solution of π-allyl–Ni(L_4)($OCOCF_3$) catalyst, which was directly used for the polymerizations without further purification.

The synthetic procedure for π-allyl–Ni(L_5)($OCOCF_3$) catalyst. The catalyst was prepared according to the reported literatures.[3] A flame-dried Schlenk flask charged with π-allyl trifluoroacetate (0.05 mL, 0.36 mmol), bis(1,5-cyclooctadiene)nickel(0) [Ni($COD)_2$, 0.10 g, 0.36 mmol], and toluene (14.0 mL) was sealed with a rubber septum. The mixture was stirred at room temperature for 20 min under dry N_2 atmosphere. Then, ligand L_5 (PPh_3, 1.0 M in toluene, 0.37

mL, 0.37 mmol) was introduced *via* a gastight syringe at 0 °C. The mixture was stirred at room temperature for 1 h to afford a solution of π-allyl–Ni(L_5)($OCOCF_3$) catalyst, which was directly used for the polymerizations without further purification.

The synthetic procedure for π-allyl–Ni(L_6)($OCOCF_3$) catalyst. The catalyst was prepared according to the reported literatures.[3] A flame-dried Schlenk flask charged with π-allyl trifluoroacetate (0.05 mL, 0.36 mmol), bis(1,5-cyclooctadiene)nickel(0) [Ni($COD)_2$, 0.10 g, 0.36 mmol], and toluene (14.0 mL) was sealed with a rubber septum. The mixture was stirred at room temperature for 20 min under dry N_2 atmosphere. Then, ligand L_6 (1,3-bis(diphenylphosphino)propane (dppp), 1.0 M in toluene, 0.37 mL, 0.37 mmol) was introduced *via* a gastight syringe at 0 °C. The mixture was stirred at room temperature for 1 h to afford a solution of π-allyl–Ni(L_6)($OCOCF_3$) catalyst, which was directly used for the polymerizations without further purification.

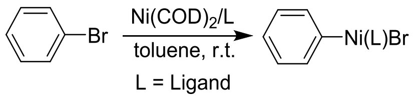

Supplementary equation 2. Synthesis of phenylnickel(II) catalysts.

The synthetic procedure for Ph–Ni(L_1)Br catalyst. The catalyst was prepared according to the reported literatures.[4] Inside a glove box, bromobenzene (15.7 mg, 0.1 mmol, 1.0 eq.) and Ming-phos (L_1) (90.3 mg, 0.2 mmol, 2.0 eq.) were placed in an oven-dried vial and were dissolved in dried toluene (1.5 mL). It was then stirred for 10 min until all starting materials were dissolved completely. Then Ni($COD)_2$ (27.5 mg, 0.1 mmol, 1.0 eq.) was added along with another 0.5 mL of dried toluene. The resulting yellow solution was allowed to stirred for another 2 h at room temperature to afford the solution of Ph–Ni(L_1)Br catalyst. This catalytic solution was directly used for diazoacetate polymerizations without further purification.

The synthetic procedure for Ph–Ni(L_2)Br catalyst. The catalyst was prepared according to the reported literatures.[4] Inside a glove box, bromobenzene (15.7 mg, 0.1 mmol, 1.0 eq.) and Xiao-phos (L_2) (81.9 mg, 0.2 mmol, 2.0 eq.) were placed in an oven-dried vial and were dissolved in dried toluene (1.5 mL). It was then stirred for 10 min until all starting materials were dissolved completely. Then Ni(COD)$_2$ (27.5 mg, 0.1 mmol, 1.0 eq.) was added along with another 0.5 mL of dried toluene. The resulting yellow solution was allowed to stirred for another 2 h at room temperature to afford the solution of Ph–Ni(L_2)Br catalyst. This catalytic solution was directly used for diazoacetate polymerizations without further purification.

The synthetic procedure for Ph–Ni(dppb)Br catalyst. The catalyst was prepared according to the reported literatures.[4] Inside a glove box, bromobenzene (15.7 mg, 0.1 mmol, 1.0 eq.) and dppb (85.3 mg, 0.2 mmol, 2.0 eq.) were placed in an oven-dried vial and were dissolved in dried toluene (1.5 mL). It was then stirred for 10 min until all starting materials were dissolved completely. Then Ni(COD)$_2$ (27.5 mg, 0.1 mmol, 1.0 eq.) was added along with another 0.5 mL of dried toluene. The resulting yellow solution was allowed to stirred for another 2 h at room temperature to afford the solution of Ph–Ni(dppb)Br catalyst. This catalytic solution was directly used for diazoacetate polymerizations without further purification.

The synthetic procedure for Ph–Ni(PPh_3)Br catalyst. The catalyst was prepared according to the reported literatures.[4] Inside a glove box, bromobenzene (15.7 mg, 0.1 mmol, 1.0 eq.) and PPh_3 (52.5 mg, 0.2 mmol, 2.0 eq.) were placed in an oven-dried vial and were dissolved in dried toluene (1.5 mL). It was then stirred for 10 min until all starting materials were dissolved completely. Then Ni(COD)$_2$ (27.5 mg, 0.1 mmol, 1.0 eq.) was added along with another 0.5 mL of dried toluene. The resulting yellow solution was allowed to stirred for another 2 h at room temperature to afford

the solution of Ph–Ni(PPh_3)Br catalyst. This catalytic solution was directly used for diazoacetate polymerizations without further purification.

The synthetic procedure for Ph–Ni(dppp)Br catalyst. The catalyst was prepared according to the reported literatures.[4] Inside a glove box, bromobenzene (15.7 mg, 0.1 mmol, 1.0 eq.) and dppp (82.5 mg, 0.2 mmol, 2.0 eq.) were placed in an oven-dried vial and were dissolved in dried toluene (1.5 mL). It was then stirred for 10 min until all starting materials were dissolved completely. Then Ni($COD)_2$ (27.5 mg, 0.1 mmol, 1.0 eq.) was added along with another 0.5 mL of dried toluene. The resulting yellow solution was allowed to stirred for another 2 h at room temperature to afford the solution of Ph–Ni(dppp)Br catalyst. This catalytic solution was directly used for diazoacetate polymerizations without further purification.

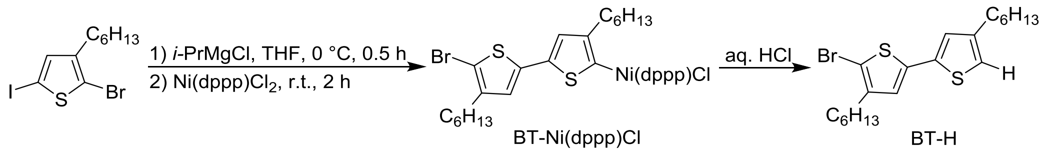

Supplementary equation 3. Synthesis of BT-Ni(dppp)Cl catalyst.

Preparation of BT-Ni(dppp)Cl catalyst: This complex was prepared according to the reported literature with modification.[5] A round-bottomed flask equipped with a three-way stopcock was heated under reduced pressure and then cooled to room temperature under a nitrogen atmosphere. 2-Bromo-3-hexyl-5-iodothiophene (372 mg, 1.0 mmol) was placed in the flask, and the atmosphere was replaced with dry nitrogen. Into the flask, dry THF (5.0 mL) was added via a syringe. The resulting mixture was stirred at 0 °C for 0.5 h. Then isopropylmagnesium chloride (*i*-PrMgCl, 2.0 M solution in THF, 0.50 mL, 1.0 mmol) was added via a syringe. After stirred at room temperature for 1 h, Ni(dppp)Cl_2 (271 mg, 0.500 mmol, 0.5 eq.) was added, and the resulting mixture was stirred at room temperature for 2 h, afforded the desired catalyst solution of BT-Ni(dppp)Cl for diazoacetates polymerization. To prove the structure, the BT-Ni(dppp)Cl catalyst

was carefully isolated and fully characterized followed a reported literature with modification.[6] The THF solution of the BT-Ni(dppp)Cl was partially evaporated under reduced pressure. Then, cold dry methanol was added, the precipitated solid was carefully collected via centrifugation at 0 °C. The isolated catalyst was dissolved in THF, precipitated in cold dry methanol, and collected *via* centrifugation. After this process was repeated three times, the catalyst was isolated and dried under vacuum overnight (344 mg, 75% yield). ^{1}H NMR (600 MHz, CD_2Cl_2, 25 °C): δ 7.60–7.57 (m, 8H, ArH of benzene ring), 7.44–7.34 (m, 13H, ArH of benzene ring and ArH of BT unit), 6.74 (s, 1H, ArH of BT unit), 2.46–2.35 (m, 4H, BT-C$\underline{H}_2$), 2.10–2.07 (m, 1H, $PCH_2CH\underline{H}$ of dppp unit), 1.83–1.77 (m, 4H, PCH_2 of dppp unit), 1.65–1.64 (m, 1H, $PCH_2C\underline{H}H$ of dppp unit), 1.51–1.48 (m, 4H, BT-$CH_2C\underline{H}_2$), 1.33–1.18 (m, 12H, BT-$CH_2CH_2(C\underline{H}_2)_3CH_3$)), 0.81–0.80 (m, 6H, CH_3 of hexyl chains). ^{13}C NMR (150 MHz, CD_2Cl_2, 25 °C) δ 143.22, 136.06, 133.52, 132.87, 131.62, 131.60, 130.62, 130.56, 128.64, 128.60, 128.57, 128.55, 128.53, 124.57, 107.67, 31.56, 30.22, 30.15, 29.75, 29.68, 29.53, 29.45, 28.81, 22.55, 14.89, 14.87, 14.84, 13.81. ^{31}P NMR (121.5 MHz, CD_2Cl_2, 25 °C) δ 30.85, 30.55. FT-IR (KBr, 25 °C, cm^{-1}): 2953 ($\nu_{C–H}$), 2924 ($\nu_{C–H}$), 2854 (ν_{C-H}). MS m/z calcd for $C_{47}H_{56}NiP_2S_2BrCl$ ($[M + H]^+$): 917.1440; Found: 917.2446. Anal. Calcd (%) for $C_{47}H_{55}NiP_2S_2BrCl$: C, 61.36%; H, 6.03%. Found: C, 61.04%; H, 6.09%.

To further confirm the structure, a portion of BT-Ni(dppp)Cl solution was quenched by aqueous hydrochloric (aq. HCl, 5%) acid and extracted with ethyl acetate. The combined organic layer was sequentially washed with water, aq. $NaHCO_3$ and brine. After dried over Na_2SO_4, it was evaporated to dryness under reduced pressure and the yielded BT-H was subjected to ^{1}H NMR, FT-IR and mass spectroscopy analyses.[7] ^{1}H NMR (400 MHz, $CDCl_3$, 25 °C): δ 7.19–7.17 (d, *J* = 8.0, ArH, 1H), 6.80–6.79 (d, *J* = 4.0, ArH, 2H), 2.58–2.52 (m, $ArCH_2$, 4H), 1.60–1.55 (m, C$\underline{H}_2$, 4H), 1.35–1.26 (m, C$\underline{H}_2$, 12H), 0.90–0.87 (m, CH_3, 6H). FT-IR (KBr, 25 °C, cm^{-1}): 2968 ($\nu_{C–H}$),

2926 ($\nu_{C–H}$), 2846 (ν_{C-H}). MS m/z calcd for $C_{20}H_{30}BrS_2$ ($[M + H]^+$): 413.10; Found: 413.11. Anal. Calcd (%) for $C_{20}H_{29}BrS_2$: C, 58.10; H, 7.07. Found: C, 57.95%; H, 7.31%.

*Procedure for diazoacetate **1a** polymerization*. Taking poly-**1a**$_{20}$ as an example. A 10 mL oven-dried flask was charged with diazoacetates **1a** (30.0 mg, 0.18 mmol), dry THF (0.36 mL), and a stir bar. After stirred at room temperature for 10 min, a solution of the BT-Ni(dppp)Cl catalyst (0.1 M, 0.09 mL, 0.009 mmol) was added to this solution *via* a microsyringe ([**1a**]$_0$/[Ni]$_0$ = 20). After the mixture solution was stirred at room temperature for 24 h, the solution was poured into large amount of ether. The precipitate solid was collected *via* filtration and dry under vacuum to afford poly-**1a**$_{20}$ as a white solid (65% yield). SEC: M_n = 2.60 kDa, M_w/M_n = 1.18. ^{1}H NMR (400 MHz, $CDCl_3$, 25 °C): δ 7.43–6.74 (br, ArH, 5H), 6.81 (s, ArH of terminal BT unit, 0.1H), 5.11–4.18 (br, OCH_2, 2H), 3.88–3.02 (br, CH, 1H), 2.53–2.49 (m, terminal thiophene-$C\underline{H_2}(CH_2)(CH_2)_3CH_3$, 0.2H), 1.65–1.50 (br, terminal thiophene-$CH_2(C\underline{H_2})(CH_2)_3CH_3$, 0.2H), 1.39–1.19 (br, terminal thiophene-$CH_2(CH_2)(C\underline{H_2})_3CH_3$, 0.6H), 0.96–0.85 (br, terminal thiophene-$CH_2(CH_2)(CH_2)_3C\underline{H_3}$, 0.3H). FT-IR (KBr, 25 °C, cm^{-1}): 2910 ($\nu_{C–H}$), 2865 ($\nu_{C–H}$), 1730 ($\nu_{C=O}$).

*Procedure for diazoacetate **1b** polymerization*. Taking poly-**1b**$_{50}$ as an example. A 10 mL oven-dried flask was charged with diazoacetates **1b** (104.5 mg, 0.45 mmol), dry THF (0.90 mL), and a stir bar. After stirred at room temperature for 10 min, a solution of the BT-Ni(dppp)Cl catalyst (0.1 M, 0.09 mL, 0.009 mmol) was added to this solution via a microsyringe ([**1b**]$_0$/[Ni]$_0$ = 50). After the mixture solution was stirred at room temperature for 24 h, the solution was poured into large amount of ether. The precipitate solid was collected via filtration and dry under vacuum to afford poly-**1b**$_{50}$ as a white solid (60% yield). SEC: M_n = 5.58 kDa, M_w/M_n = 1.17. ^{1}H NMR (400 MHz, $CDCl_3$, 25 °C): δ 6.82 (s, ArH of terminal BT unit, 0.04H), 2.43–2.31 (m, terminal thiophene-$C\underline{H_2}$, 0.08H), 4.17 (br, CO_2CH_2, 2H), 3.61–3.52 (br, $CH_2(CH_2CH_2O)_2$, 10H), 3.35 (br, CH and CH_3,

4H), 1.23–0.86 (br, terminal thiophene-CH_2 and CH_3, 0.44H). FT-IR (KBr, 25 °C, cm^{-1}): 2877 ($\nu_{C–H}$), 1734 ($\nu_{C=O}$).

*Procedure for diazoacetate **1c** polymerization*. Taking poly-**1c**$_{50}$ as an example. A 10 mL oven-dried flask was charged with diazoacetates **1c** (51.5 mg, 0.45 mmol), dry THF (0.90 mL), and a stir bar. After stirred at room temperature for 10 min, a solution of the BT-Ni(dppp)Cl catalyst (0.1 M, 0.09 mL, 0.009 mmol) was added to this solution via a microsyringe ($[\mathbf{1c}]_0/[Ni]_0 = 50$). After the mixture solution was stirred at room temperature for 24 h, the solution was poured into large amount of ether. The precipitate solid was collected via filtration and dry under vacuum to afford poly-**1c**$_{50}$ as a white solid (66% yield). SEC: M_n = 4.85 kDa, M_w/M_n = 1.19. ^{1}H NMR (400 MHz, $CDCl_3$, 25 °C): δ 6.82 (s, ArH of terminal BT unit, 0.04H), 4.08 (br, CO_2CH_2, 2H), 3.18 (br, CH of main chain, 1H), 2.53–2.31 (m, terminal thiophene-CH_2, 0.08 H), 1.24–0.86 (br, CH_3 and terminal thiophene-CH_2 and CH_3, 3.44H). FT-IR (KBr, 25 °C, cm^{-1}): 2974, 2900, 2883 ($\nu_{C–H}$), 1742 ($\nu_{C=O}$).

*Procedure for diazoacetate **1d** polymerization*. Taking poly-**1d**$_{50}$ as an example. A 10 mL oven-dried flask was charged with diazoacetates **1d** (76.6 mg, 0.45 mmol), dry THF (0.90 mL), and a stir bar. After stirred at room temperature for 10 min, a solution of the BT-Ni(dppp)Cl catalyst (0.1 M, 0.09 mL, 0.009 mmol) was added to this solution via a microsyringe ($[\mathbf{1d}]_0/[Ni]_0 = 50$). After the mixture solution was stirred at room temperature for 24 h, the solution was poured into large amount of ether. The precipitate solid was collected via filtration and dry under vacuum to afford poly-**1d**$_{50}$ as a white solid (62% yield). SEC: M_n = 6.81 kDa, M_w/M_n = 1.18. ^{1}H NMR (400 MHz, $CDCl_3$, 25 °C): δ 6.82 (s, ArH of terminal BT unit, 0.04 H), 4.64 (br, CO_2CH_2, 2H), 3.16–2.30 (br, CH of main chain and terminal thiophene-CH_2, 1H), 2.16–1.85 (br, $OCH_2C\underline{H_2}$, 2H), 1.85–1.61 (br,

CH_2, 6H), 1.27–1.04 (br, CH_3, 3H). FT-IR (KBr, 25 °C, cm^{-1}): 2922 (ν_{C-H}), 2831 (ν_{C-H}), 1730 ($\nu_{C=O}$).

Procedure for kinetic studies. A mixture of monomer **1a** (100.0 mg, 0.56 mmol) and a standard polystyrene (PSt, M_n = 41.4 kDa, M_w/M_n =1.02, 20.0 mg) were placed in a dry ampule, which was then evacuated on a vacuum line and flushed with dry nitrogen. After the evacuation-flush procedure had been repeated three times, a three-way stopcock was attached to the ampule, and dry THF (1.10 mL) was added by a microsyringe. To this was added a solution of the BT-Ni(dppp)Cl in THF (56.0 μL) *via* a microsyringe at ambient temperature ([**1a**]$_0$ = 0.5 M, [**1a**]$_0$/[Ni]$_0$ = 100). The mixture was then stirred under a dry nitrogen atmosphere at room temperature. The polymerization of **1a** was followed by measuring SEC of the aliquots removed from the reaction mixture at appropriate time intervals. The peak area of unreacted **1a** relative to that of the internal PSt standard was used for the determination of the conversion of **1a** on the basis of the linear calibration curve. The M_n and M_w/M_n were estimated by SEC and reported as equivalent to polystyrene standards.

Synthetic procedure for P3HT homopolymer and the block copolymer. Taking poly(**2**$_{20}$-*b*-**1a**$_{40}$) as an example. A 10 mL oven-dried flask was charged with 2-bromo-3-hexyl-5-iodothiophene (74 mg, 0.20 mmol), dry THF (2.0 mL) and a stir bar. After a solution of isopropylmagnesium chloride (2.0 M solution in THF, 0.10 mL, 0.20 mmol) was added via a syringe, the resulting mixture was stirred at 25 °C for 2 h. Then Ni(dppp)Cl_2 (5.4 mg, 0.01 mmol) was added to the reaction mixture under dry N_2 atmosphere ([**2**]$_0$/[Ni(dppp)Cl_2]$_0$ = 20). The resulting solution was stirred for further 2 h to generated the Ni(II)-terminated P3HT homopolymer. When SEC analyses revealed the M_n of the generated polymer ceased to increase, that is the monomer **2** was nearly completely consumed, monomer **1a** (100.0 mg, 0.60 mmol) was added to the polymerization solution of in-

situ generated Ni(II)-terminated P3HT (poly-$\mathbf{2}_{20}$, M_n = 6.71 kDa, M_w/M_n = 1.19). The feed ratio of monomer **1a** to the Ni complex was 40. The reaction was stirred at room temperature for further 24 h. The polymerization solution was then precipitated into a large amount of ether, the precipitated solid was collected by centrifugation, and dried in vacuum at room temperature overnight, afforded poly($\mathbf{2}_{20}$-*b*-$\mathbf{1a}_{40}$) as a dark-purple solid (86 mg, 71% yield over two steps). SEC: M_n = 12.6 kDa, M_w/M_n = 1.18. ^{1}H NMR (400 MHz, $CDCl_3$, 25 °C): δ 7.21–6.79 (br, ArH of poly-$\mathbf{2}_{20}$ and poly-$\mathbf{1a}_{40}$ segment, 5.5H), 4.93–4.24 (br, OCH_2 of poly-$\mathbf{1a}_{40}$ block, 2H), 3.87–3.01 (br, CH of poly-$\mathbf{1a}_{40}$ main chain, 1H), 2.81–2.61 (br, thiophene-CH_2, 1H), 1.72–0.92 (br, CH_2 and CH_3 of poly-$\mathbf{2}_{20}$ segment, 5.5H). FT-IR (KBr, 25 °C, cm^{-1}): 2910 ($\nu_{C–H}$), 2865 ($\nu_{C–H}$), 1730 ($\nu_{NHC=O}$).

*Synthetic procedure for Poly(**2**20-b-**1b**40).* A 10 mL oven-dried flask was charged with 2-bromo-3-hexyl-5-iodothiophene (74 mg, 0.20 mmol), dry THF (2.0 mL) and a stir bar. After a solution of isopropylmagnesium chloride (2.0 M solution in THF, 0.10 mL, 0.20 mmol) was added via a syringe, the resulting mixture was stirred at 25 °C for 2 h. Then Ni(dppp)Cl_2 (5.4 mg, 0.01 mmol) was added to the reaction mixture under dry N_2 atmosphere ([**2**]$_0$/[Ni(dppp)Cl_2]$_0$ = 20). The resulting solution was stirred for further 2 h to generated the Ni(II)-terminated P3HT homopolymer. When SEC analyses revealed the M_n of the generated polymer ceased to increase, that is the monomer **2** was nearly completely consumed, monomer **1b** (139 mg, 0.60 mmol) was added to the polymerization solution of in-situ generated Ni(II)-terminated P3HT (poly-$\mathbf{2}_{20}$, M_n = 6.71 kDa, M_w/M_n = 1.19). The feed ratio of monomer **1b** to the Ni complex was 40. The reaction was stirred at room temperature for further 24 h. The polymerization solution was then precipitated into a large amount of ether, the precipitated solid was collected by centrifugation, and dried in vacuum at room temperature overnight, afforded poly($\mathbf{2}_{20}$-*b*-$\mathbf{1b}_{40}$) as a dark-purple solid (112 mg, 72% yield over two steps). SEC: M_n = 10.1 kDa, M_w/M_n = 1.14. ^{1}H NMR (400 MHz, $CDCl_3$, 25 °C): δ 6.97 (br, ArH of poly-$\mathbf{2}_{20}$ segment, 1H), 4.35 (br, CO_2CH_2 of poly-$\mathbf{1b}_{40}$ segment, 4H), 3.76–3.56

(br, OCH_2 of poly-**1b**$_{40}$ segment, 20H), 3.37 (br, CH of poly-**1b**$_{40}$ main chain and CH_3 of poly-**1b**$_{40}$ side chain, 8H), 2.78–0.88 (br, CH_2 and CH_3 of poly-**2**$_{20}$, 11H). FT-IR (KBr, 25 °C, cm^{-1}): 2877 ($\nu_{C–H}$), 1734 ($\nu_{C=O}$).

AFM measurements. Two stock solutions of poly(**2**$_{20}$-*b*-**1b**$_{40}$) in dry THF and IPA (0.3 mg/mL) were prepared and aged for 12 h at room temperature. Samples for AFM measurements were prepared by casting 20 μL aliquots of the stock solutions on silicon wafers. The samples on silicon wafers were annealed under the respective solvent vapor atmosphere at room temperature for 12 h, and then dried under vacuum for 2 h. Then the samples on silicon wafers were subjected to AFM measurements.

TEM measurements. Two stock solutions of poly(**2**$_{20}$-*b*-**1b**$_{40}$) in dry THF and IPA (0.3 mg/mL) were prepared and aged at room temperature for 12 h. The samples for TEM observation were prepared by placing 10 μL of the stock solutions on copper grids coated with thin film and carbon. After the redundant solution on the copper grids was removed by filter paper, the samples were dried at room temperature for 2 h and then subjected to TEM analyses.

DLS analyses: A solution of poly(**2**$_{20}$-*b*-**1b**$_{40}$) in IPA (0.3 mg/mL) was prepared and aged at room temperature for 12 h, and then subjected to DLS analyses. All data were averaged over three times measurements.

DFT computational details. The geometry optimizations were performed with B3LYP implemented in the Gaussian 16 packages in gas phase with TZVP basis set. Frequency calculations were performed to confirm that zero imaginary frequencies for all optimized intermediates and only one imaginary frequencies for transition states. Intrinsic reaction coordinate (IRC) calculations were performed for the rate-determining step transition states to confirm that the potential energy surface. To save computational costs, “g09defaults” keyword was used in all calculations.

Supplementary Table 1. Results for the polymerizations using BT–Ni(dppp)Cl catalyst.

run[a]	Monomer	Solvent	Temp.	[**1**]$_0$/[Ni]$_0$	M_n[b] (kDa)	M_w/M_n[b]	Yield[c]
1	**1a**	THF	r.t.	30	3.25	1.12	63%
2	**1a**	THF	r.t.	35	3.76	1.20	68%
3	**1a**	THF	r.t.	40	4.10	1.12	65%
4	**1a**	THF	r.t.	45	4.58	1.14	62%
5	**1a**	Toluene	r.t.	50	2.82	1.23	33%
6	**1a**	CH_2Cl_2	r.t.	50	2.71	1.13	24%
7	**1a**	THF	r.t.	50	5.10	1.15	55%
8	**1a**	$CHCl_3$	r.t.	50	--[d]	--[d]	15%
9	**1a**	THF	0°C	50	3.50	1.23	58%
10	**1a**	THF	55°C	50	2.71	1.26	50%
11	**1b**	THF	r.t	30	3.42	1.18	63%
12	**1b**	THF	r.t.	50	5.58	1.17	60%
13	**1c**	THF	r.t.	50	4.85	1.19	66%
14	**1d**	THF	r.t.	50	6.81	1.18	62%

[a]The polymers were prepared on the basis of Scheme 1. [b]M_n and M_w/M_n were determined by SEC.

[c]The isolated yields. [d]M_n was lower than the detection limit of SEC.

Supplementary Table 2. Energies of all the DFT optimized minima and transition states as presented in the main text and negative eigenvalue frequencies for all the transition states.

Species	Electronic Energies (E, in Hartree)	Gibbs Free Energies (G_{298}, in Hartree)	Negative Eigenvalues	Relative Electronic Energies (ΔE, in kcal/mol)	Relative Gibbs Free Energies (ΔG_{298}, in kcal/mol)
**BDA**	-607.888628	-607.771976	-	0.0	0.0
**N_2**	-109.567199	-109.580017	-	0.0	0.0
**Ph-IN1**	-6041.980059	-6041.508655	-	0.0	0.0
**Ph-TS1**	-6649.844955	-6649.234974	-64.45	14.9	28.7
**Ph-IN2**	-6649.848296	-6649.239836	-	12.8	25.6
**Ph-TS2**	-6649.825432	-6649.218567	-405.78	27.1	38.9
**Ph-IN3**	-6649.959366	-6649.353367	-	-56.9	-45.6
**Ph-IN4**	-6540.393618	-6539.782746	-	-57.8	-51.5
**BT-IN1**	-7846.165943	-7845.384662	-	0.0	0.0
**BT-TS2**	-8454.013326	-8453.095841	-464.79	25.9	38.2
**BT-IN4**	-8344.569115	-8343.651504	-	-51.3	-47.0

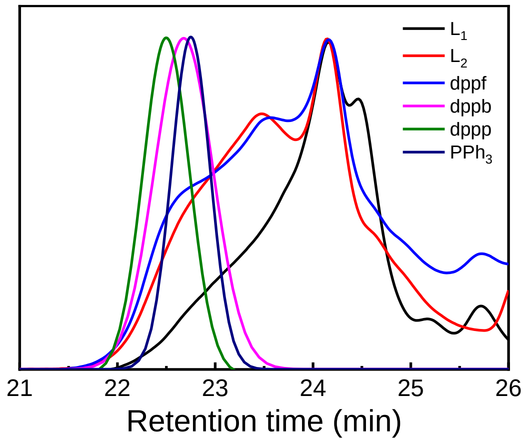

Supplementary Fig. 1 Size exclusion chromatograms of poly-**1a**$_{m}$ using phenylnickel(II) catalysts with different ligands.

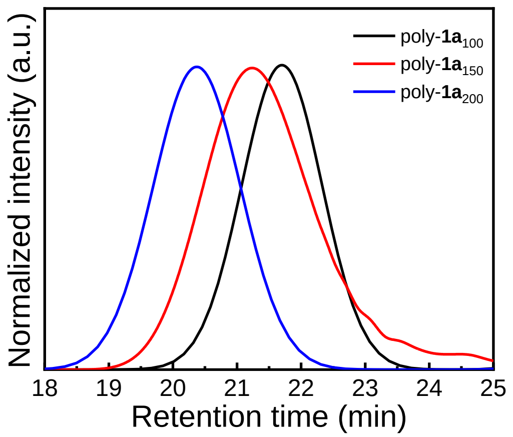

Supplementary Fig. 2 Size exclusion chromatograms of poly-**1a**$_{100}$, poly-**1a**$_{150}$, and poly-**1a**$_{200}$ prepared using BT-Ni(dppp)Cl as catalyst in THF at room temperature.

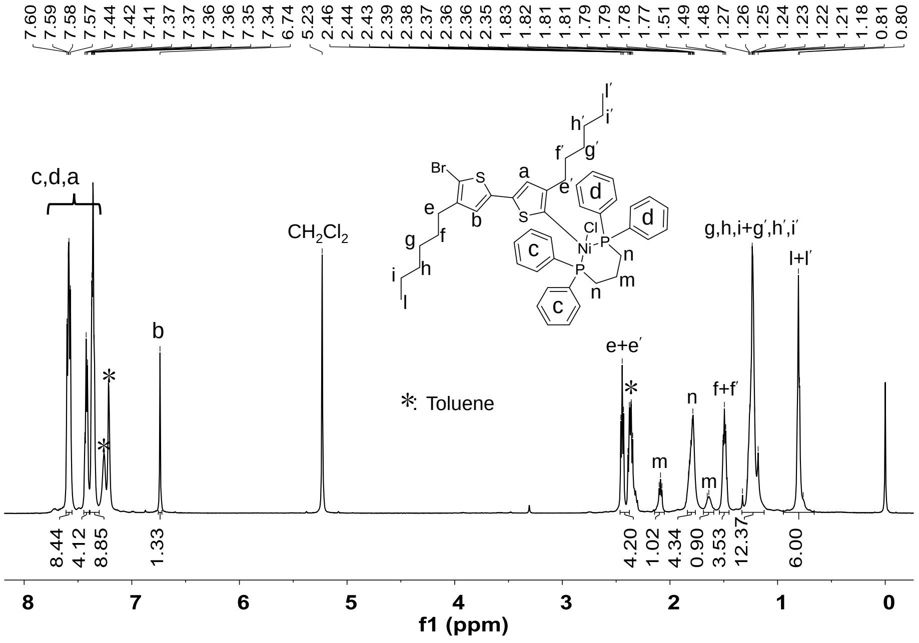

Supplementary Fig. 3 ^{1}H NMR (600 MHz) spectrum of BT-Ni(dppp)Cl measured in CD_2Cl_2 at 25 °C.

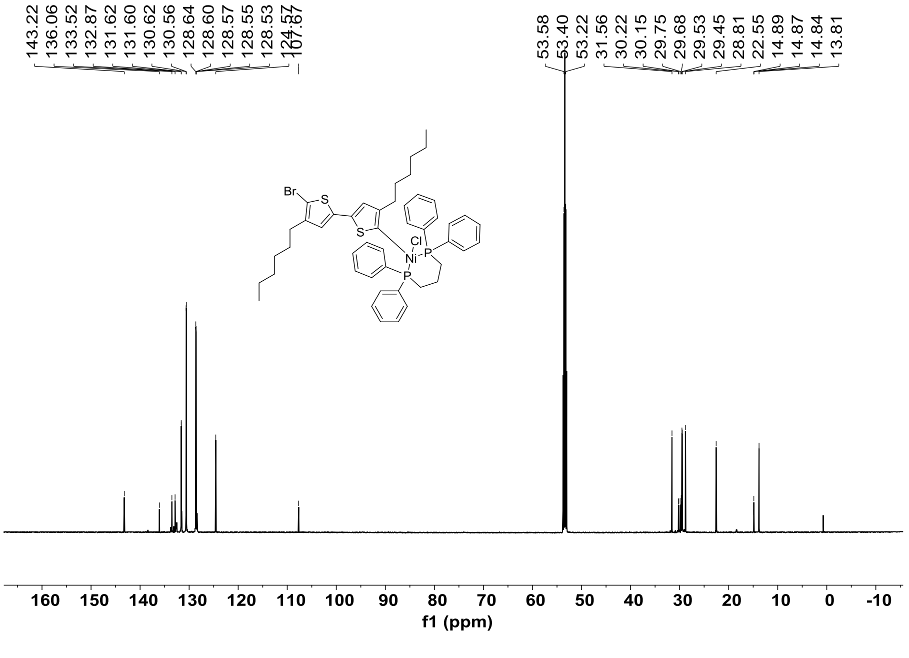

Supplementary Fig. 4 ^{13}C NMR (150 MHz) spectrum of BT-Ni(dppp)Cl measured in $CDCl_3$ at 25 °C.

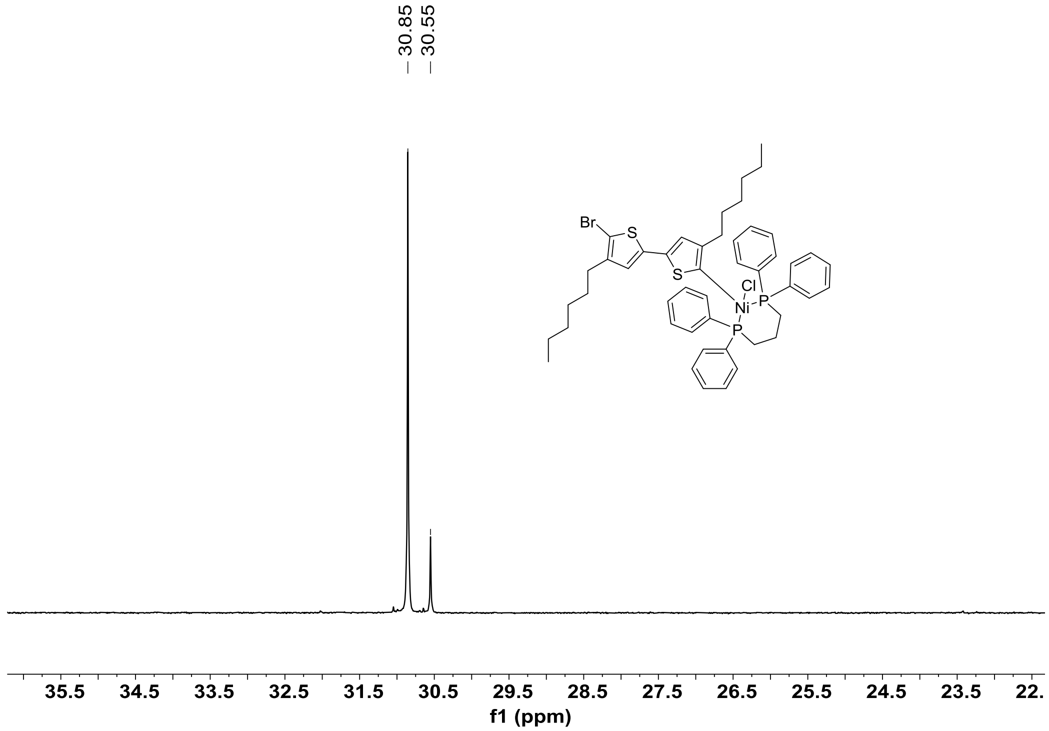

Supplementary Fig. 5 ^{31}P NMR (121.5 MHz) spectrum of BT-Ni(dppp)Cl measured in $CDCl_3$ at 25 °C.

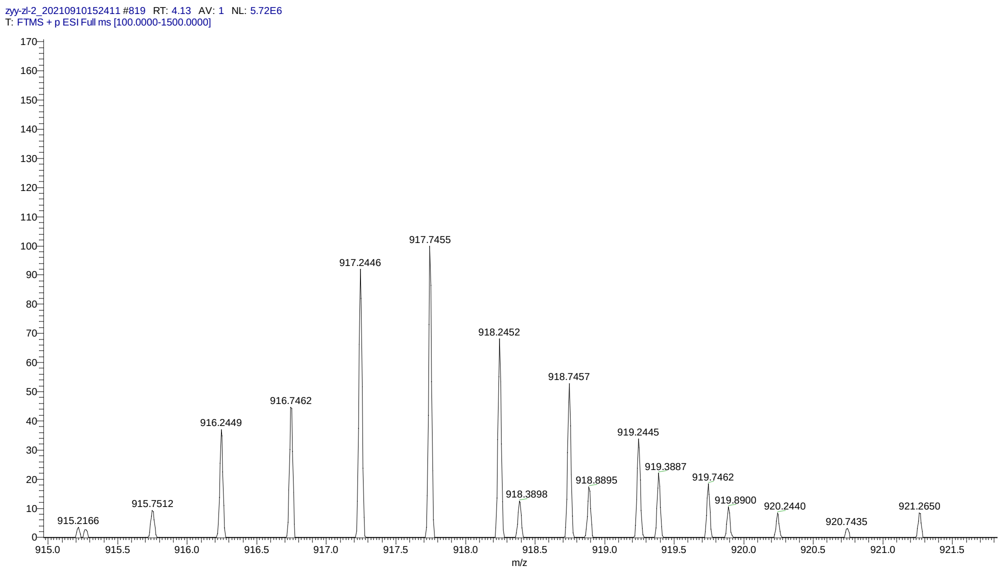

Supplementary Fig. 6 MALDI-TOF spectrum of BT-Ni(dppp)Cl.

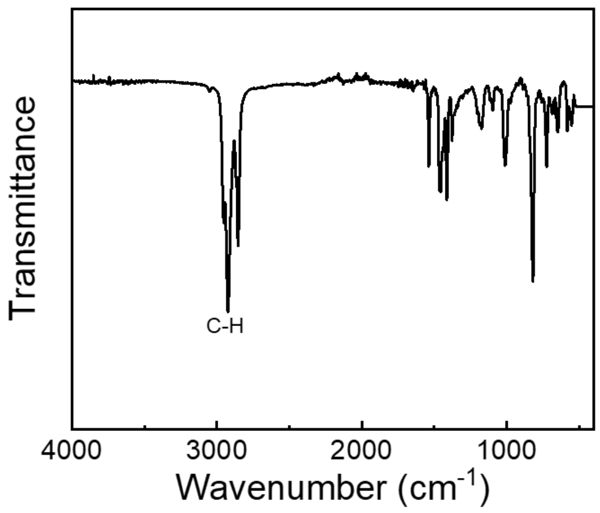

Supplementary Fig. 7 FT-IR spectrum of BT-Ni(dppp)Cl measured at 25 °C using KBr pellets.

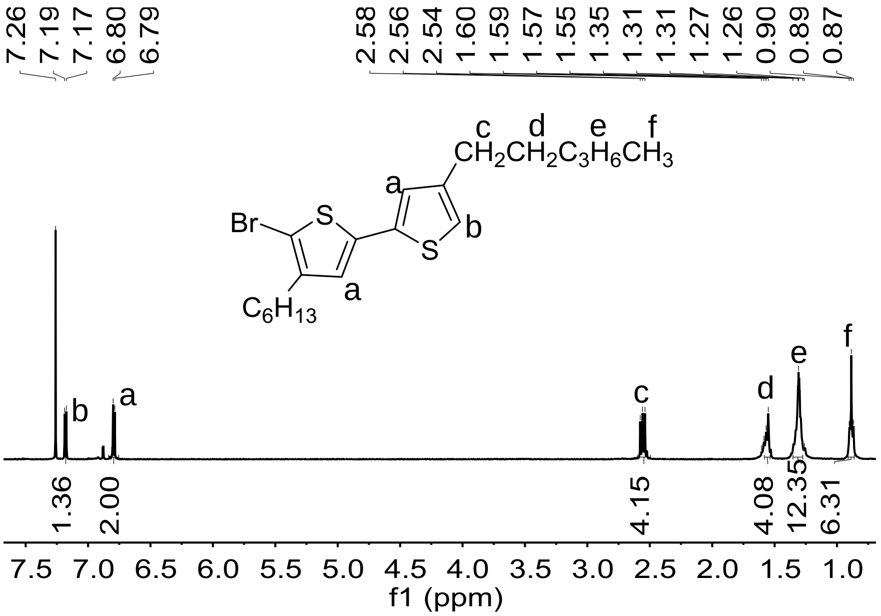

Supplementary Fig. 8 ^{1}H NMR (400 MHz) spectrum of BT-H measured in $CDCl_3$ at 25 °C.

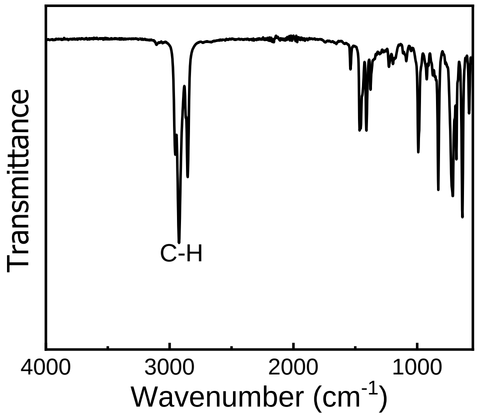

Supplementary Fig. 9 FT-IR spectrum of BT-H using KBr pellets.

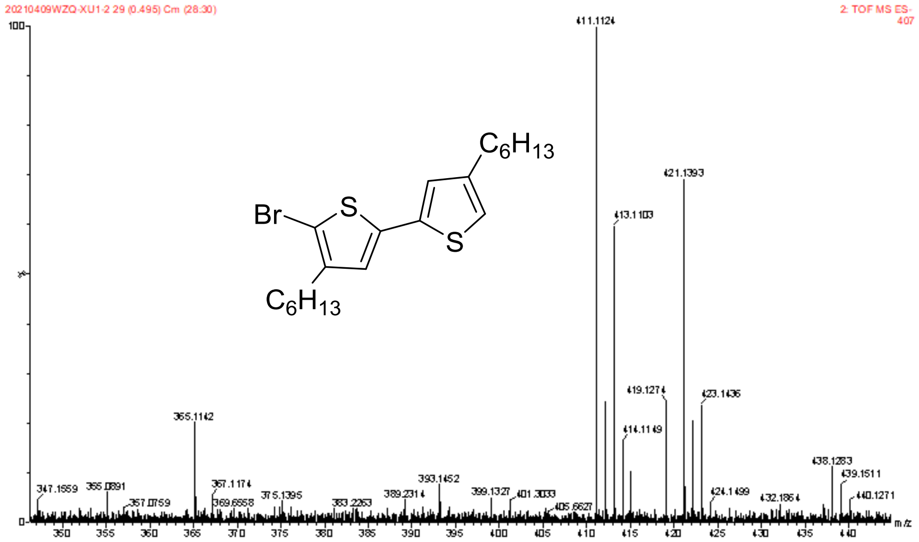

Supplementary Fig. 10 Electrospray ionization (ESI) mass spectrum of BT-H.

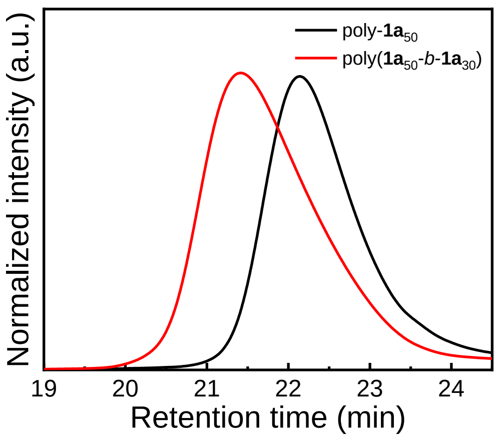

Supplementary Fig. 11 Size exclusion chromatograms of macroinitiator poly-**1a**$_{50}$ and the resulting chain extended poly(**1a**$_{50}$-*b*-**1a**$_{30}$) using BT-Ni(dppp)Cl as catalyst.

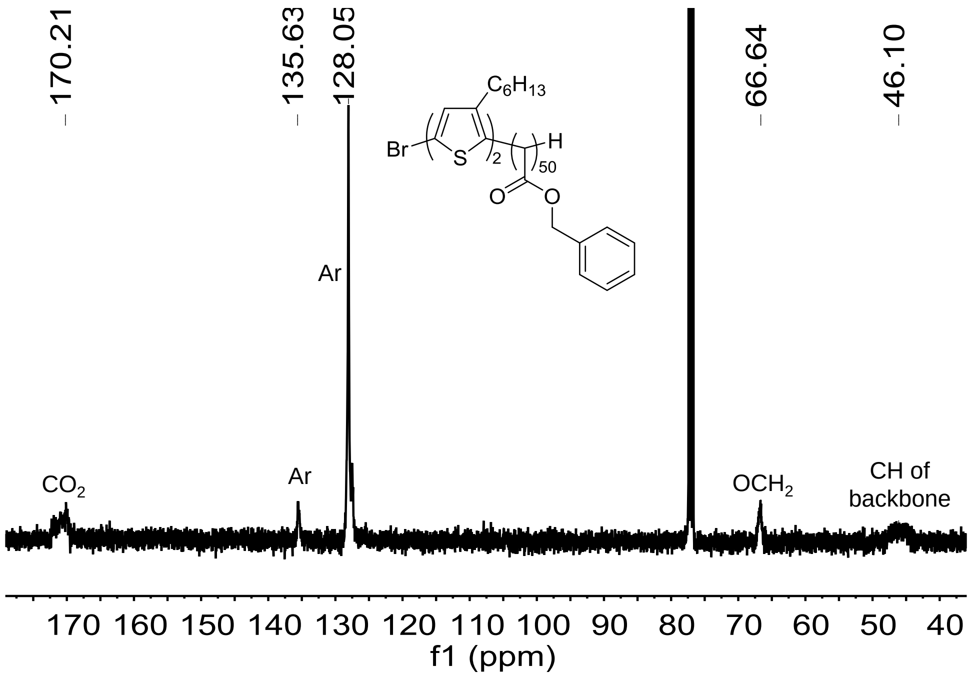

Supplementary Fig. 12 ^{13}C NMR (150 MHz) spectrum of poly-**1a**$_{50}$ measured in $CDCl_3$ at room temperature.

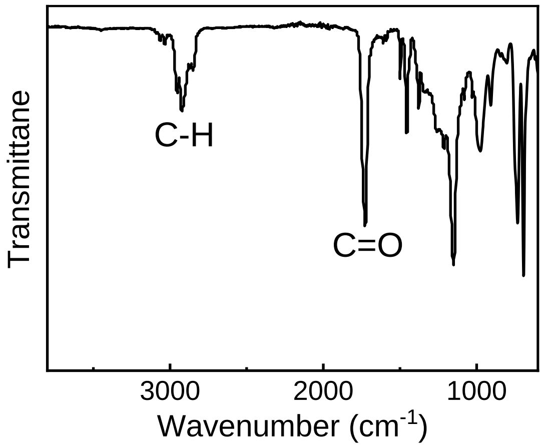

Supplementary Fig. 13 FT-IR spectrum of poly-**1a**$_{50}$ measured at 25 °C using KBr pellets.

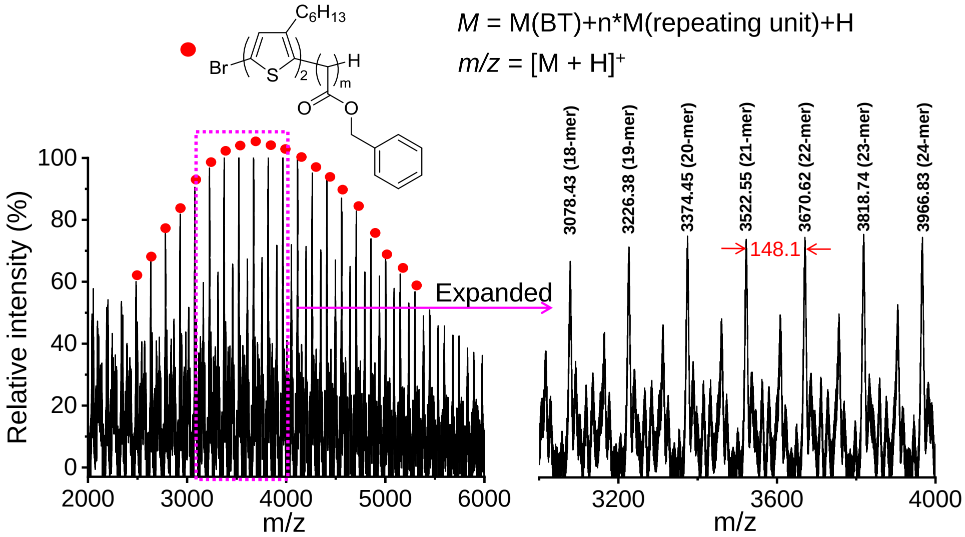

Supplementary Fig. 14 MALDI-TOF mass spectrum of poly-**1a**$_{20}$.

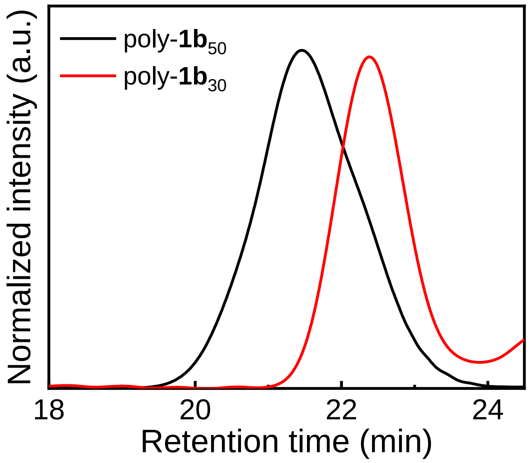

Supplementary Fig. 15 Size exclusion chromatograms of poly-**1b**$_{30}$ and poly-**1b**$_{50}$ prepared via the polymerization of **1b** using BT-Ni(dppp)Cl as catalyst.

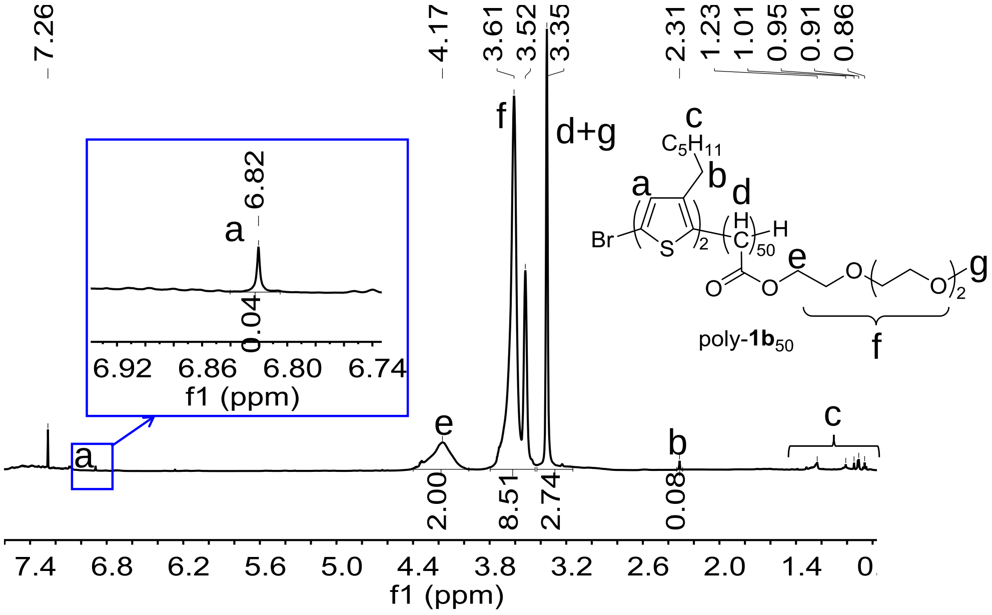

Supplementary Fig. 16 ^{1}H NMR (400 MHz) spectrum of poly-**1b**$_{50}$ measured in $CDCl_3$ at room temperature.

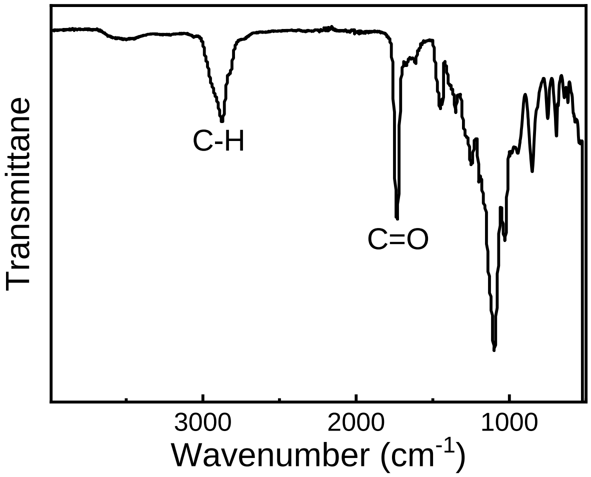

Supplementary Fig. 17 FT-IR spectrum of poly-**1b**$_{50}$ measured at 25 °C using KBr pellets.

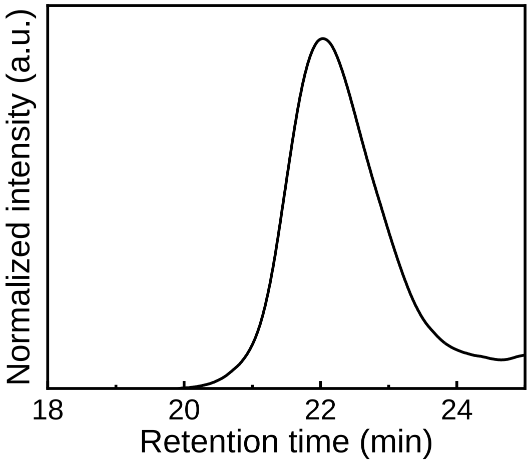

Supplementary Fig. 18 Size exclusion chromatograms of poly-**1c**$_{50}$ prepared using BT-Ni(dppp)Cl as catalyst.

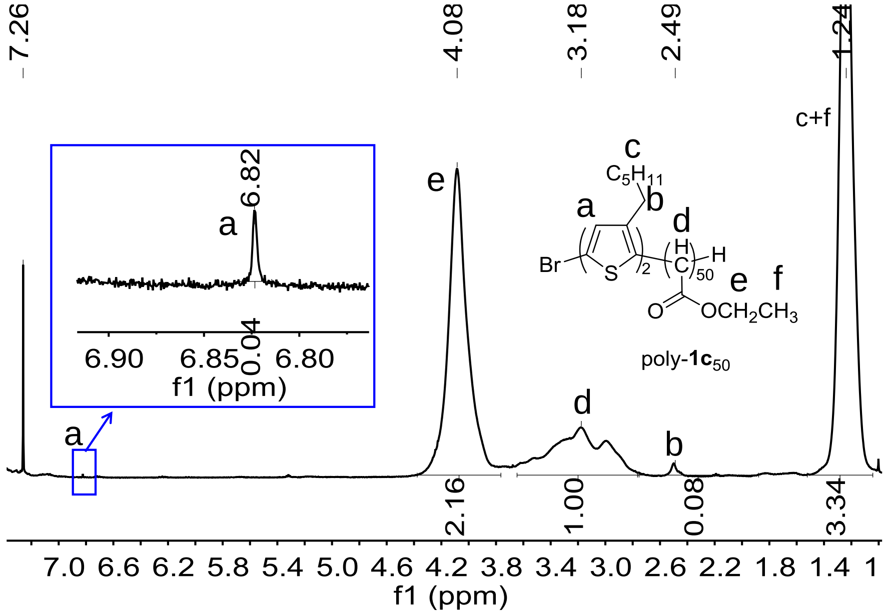

Supplementary Fig. 19 ^{1}H NMR (400 MHz) spectrum of poly-**1c**$_{50}$ measured in $CDCl_3$ at room temperature.

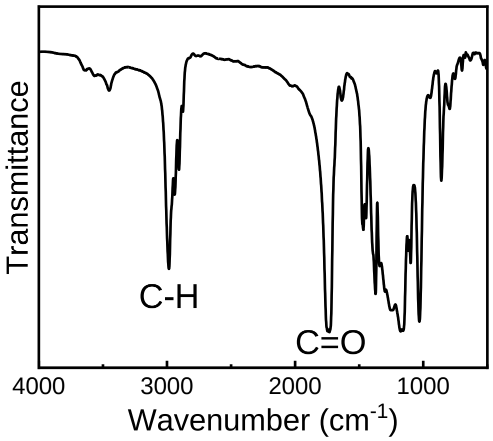

Supplementary Fig. 20 FT-IR spectrum of poly-**1c**$_{50}$ measured at 25 °C using KBr pellets.

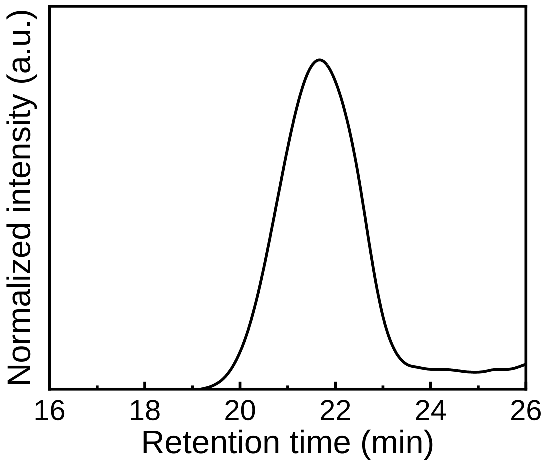

Supplementary Fig. 21 Size exclusion chromatograms of poly-**1d**$_{50}$ prepared using BT-Ni(dppp)Cl as catalyst.

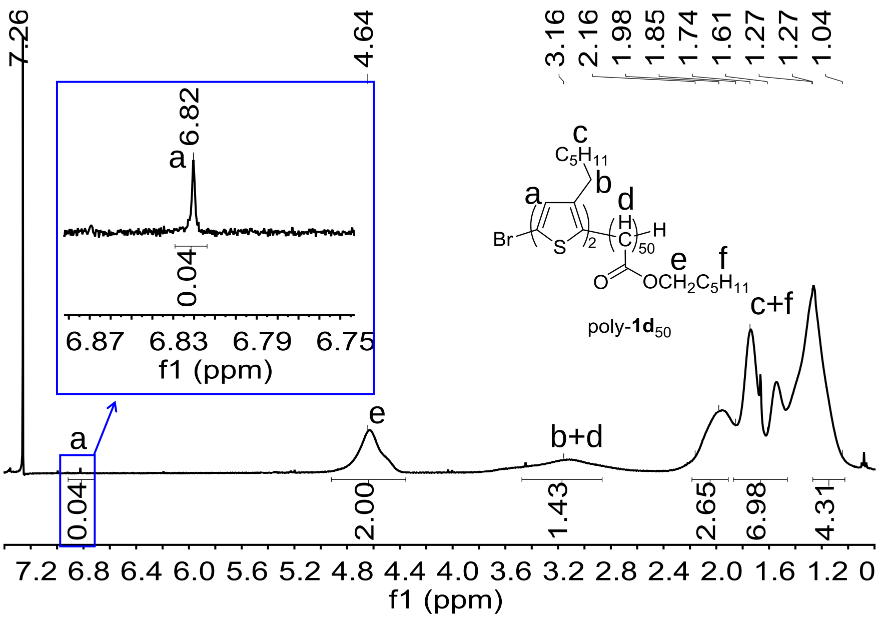

Supplementary Fig. 22 ^{1}H NMR (400 MHz) spectrum of poly-**1d**$_{50}$ measured in $CDCl_3$ at room temperature.

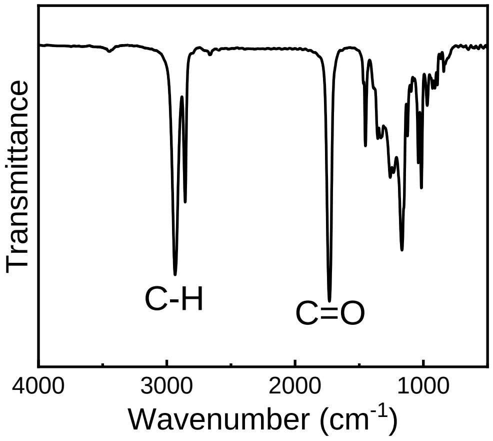

Supplementary Fig. 23 FT-IR spectrum of poly-**1d**$_{50}$ measured at 25 °C using KBr pellets.

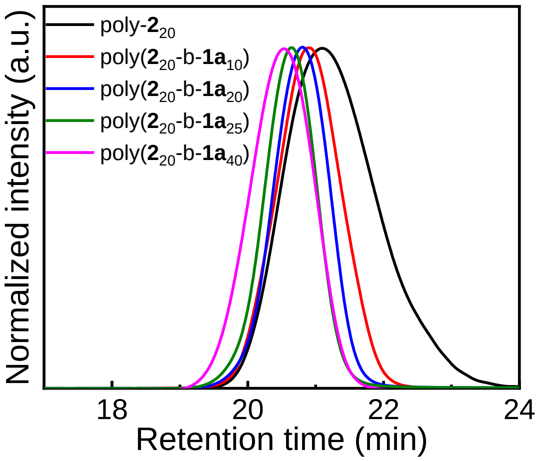

Supplementary Fig. 24 Size exclusion chromatograms of poly(**2**$_{20}$-*b*-**1a**$_{n}$)s prepared from the copolymerization of **1a** using Ni-terminated poly-**2**$_{20}$ (M_n = 6.7 kDa, M_w/M_n = 1.19) as a common macroinitiator in THF at room temperature with the initial feed ratio of **1a** to the Ni(II) complex were 10, 20, 25, and 40.

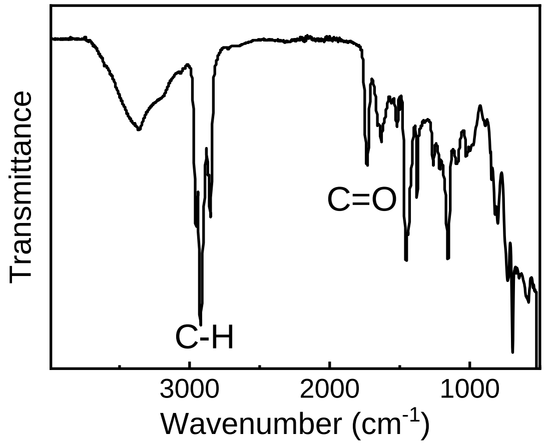

Supplementary Fig. 25 FT-IR spectrum of poly($\mathbf{2}_{20}$-*b*-$\mathbf{1a}_{40}$) measured at 25 °C using KBr pellets.

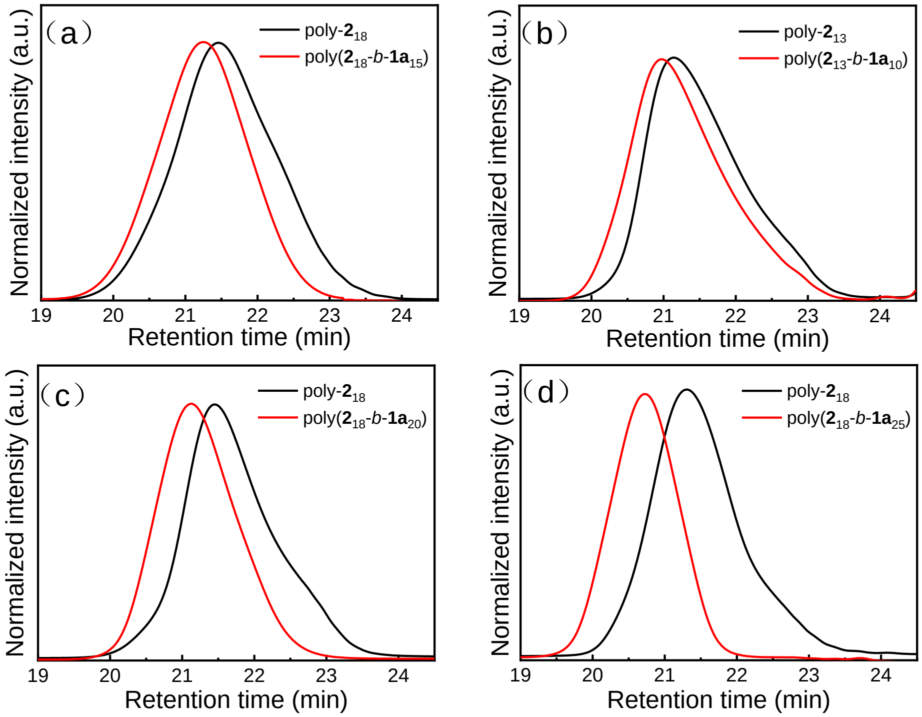

Supplementary Fig. 26 SEC of poly-$\mathbf{2}_{18}$ and the resulting poly($\mathbf{2}_{18}$-*b*-$\mathbf{1a}_{15}$) (a), poly-$\mathbf{2}_{13}$ and the resulting poly($\mathbf{2}_{13}$-*b*-$\mathbf{1a}_{10}$) (b), poly-$\mathbf{2}_{18}$ and the resulting poly($\mathbf{2}_{18}$-*b*-$\mathbf{1a}_{20}$) (c), poly-$\mathbf{2}_{18}$ and the resulting poly($\mathbf{2}_{18}$-*b*-$\mathbf{1a}_{25}$) (d) measured in THF at 25 °C.

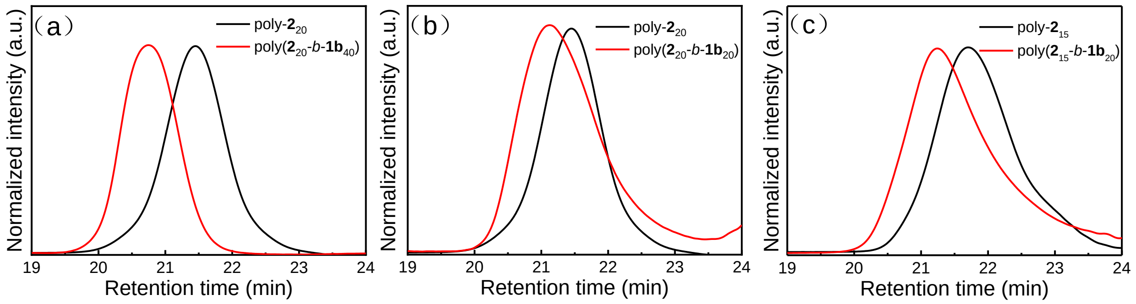

Supplementary Fig. 27 SEC of poly-$\mathbf{2}_{20}$ and the resulting poly($\mathbf{2}_{20}$-*b*-$\mathbf{1b}_{40}$) (a), poly-$\mathbf{2}_{20}$ and the resulting poly($\mathbf{2}_{20}$-*b*-$\mathbf{1b}_{20}$) (b), poly-$\mathbf{2}_{15}$ and the resulting poly($\mathbf{2}_{15}$-*b*-$\mathbf{1b}_{20}$) (c) measured in THF at 25 °C.

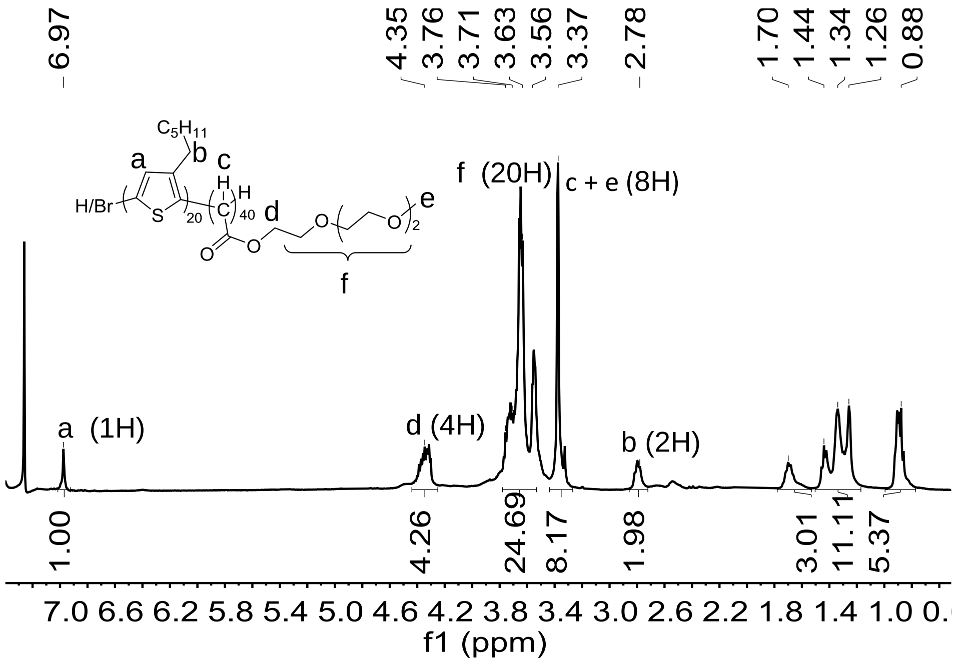

Supplementary Fig. 28 ^{1}H NMR (400 MHz) spectrum of poly($\mathbf{2}_{20}$-*b*-$\mathbf{1b}_{40}$) measured in $CDCl_3$ at room temperature.

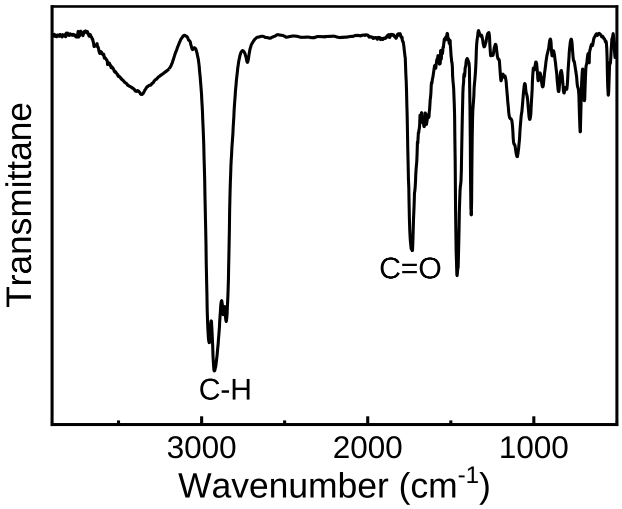

Supplementary Fig. 29 FT-IR spectrum of poly($\mathbf{2}_{20}$-*b*-$\mathbf{1b}_{40}$) measured at 25 °C using KBr pellets.

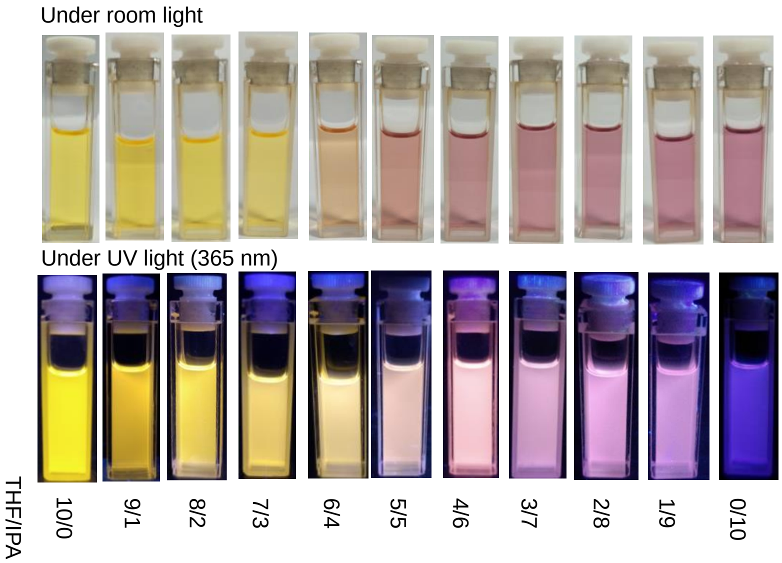

Supplementary Fig. 30 Photographs of poly($\mathbf{2}_{20}$-*b*-$\mathbf{1b}_{40}$) in THF/IPA mixtures with different volume ratios under room light and under UV 365 nm light.

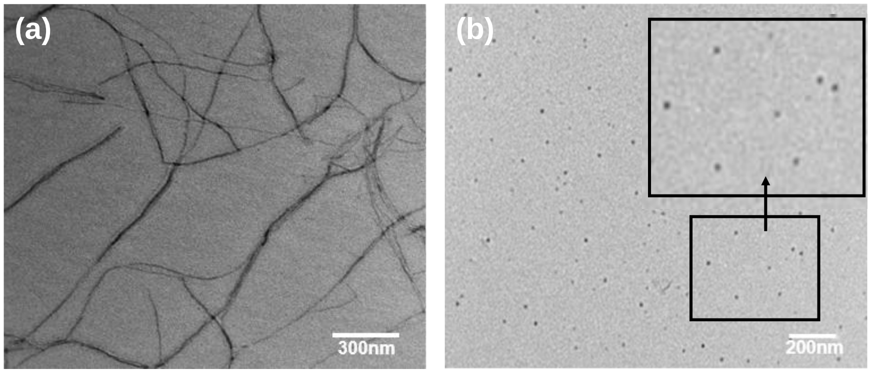

Supplementary Fig. 31 TEM images of the samples casted from poly(**2**$_{20}$-*b*-**1b**$_{40}$) solutions in THF (a) and IPA (b) at room temperature.

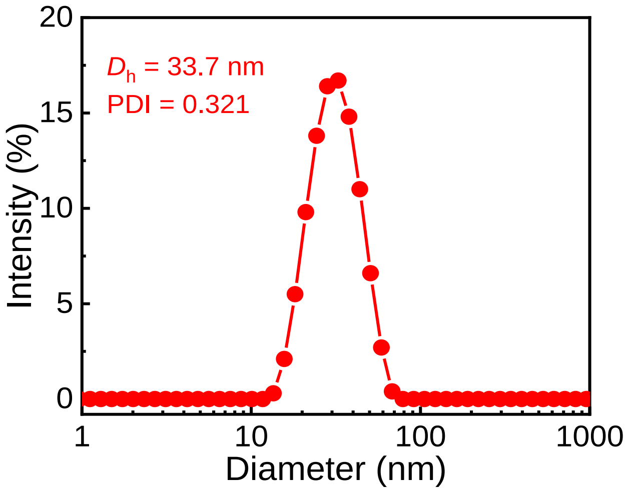

Supplementary Fig. 32 DLS curve for poly(**2**$_{20}$-*b*-**1b**$_{40}$) recorded in IPA at 25 °C (c = 0.3 mg/mL).

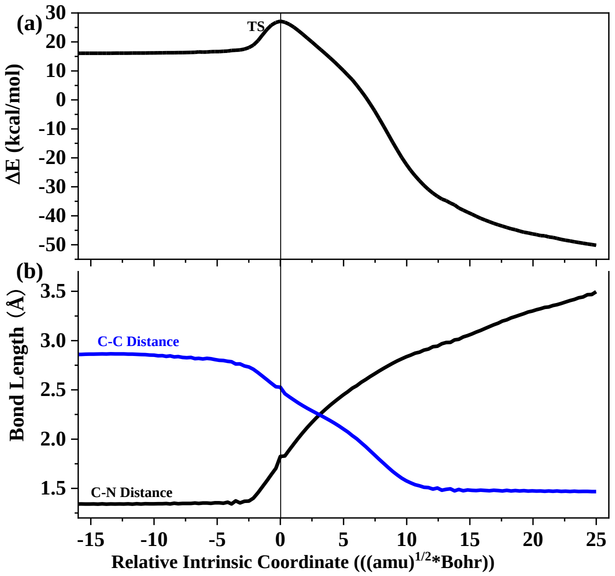

Supplementary Fig. 33 IRC calculation results for **Ph-TS2**, calculated at B3LYP/TZVP level in gas phase. Relative electronic energy in kcal/mol with **Ph-IN1** as reference point.

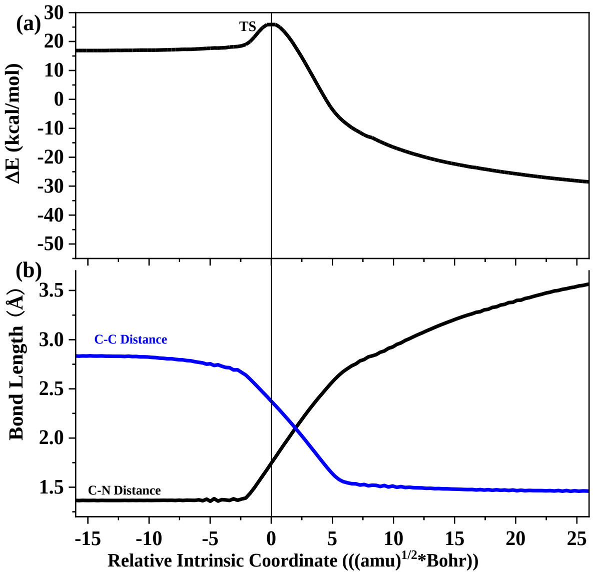

Supplementary Fig. 34 IRC calculation results for **BT-TS2**, calculated at B3LYP/TZVP level in gas phase. Relative electronic energy in kcal/mol with **BT-IN1** as reference point.

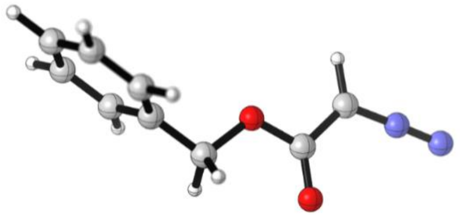

Supplementary Fig. 35 DFT optimized geometry of **1a**.

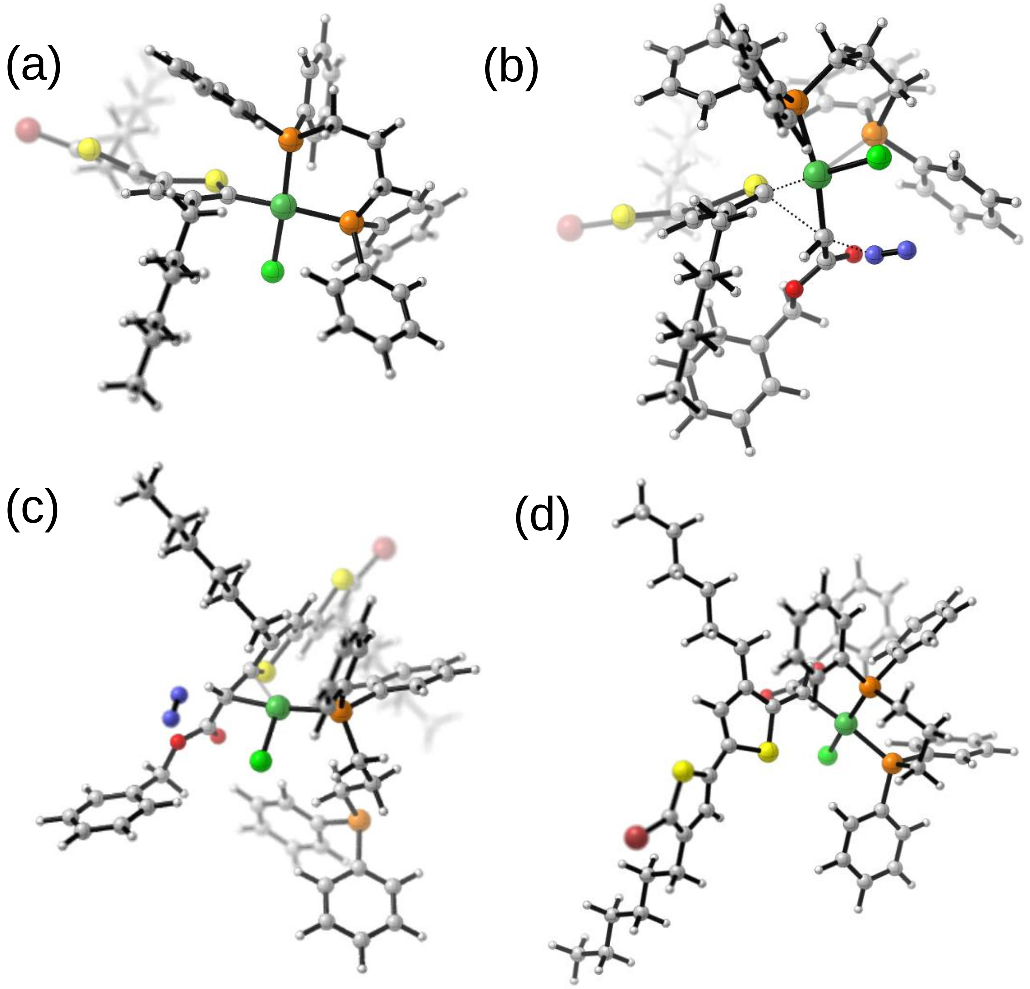

Supplementary Fig. 36 DFT optimized geometry of **BT-IN1** (a), **BT-TS2** (b), **BT-IN3** and **BT-IN4** (c).

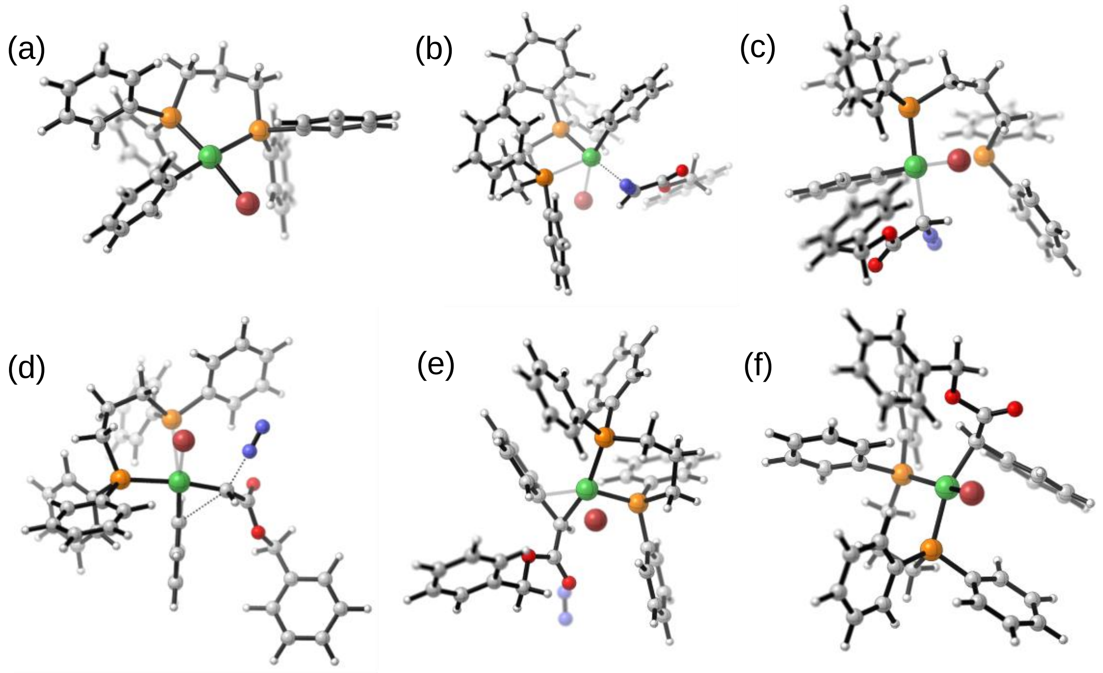

Supplementary Fig. 37 DFT optimized geometry of **Ph-IN1** (a), **Ph-TS1** (b), **Ph-IN2** (c), **Ph-TS2** (d), **Ph-IN3** (e), and **Ph-IN4** (f).

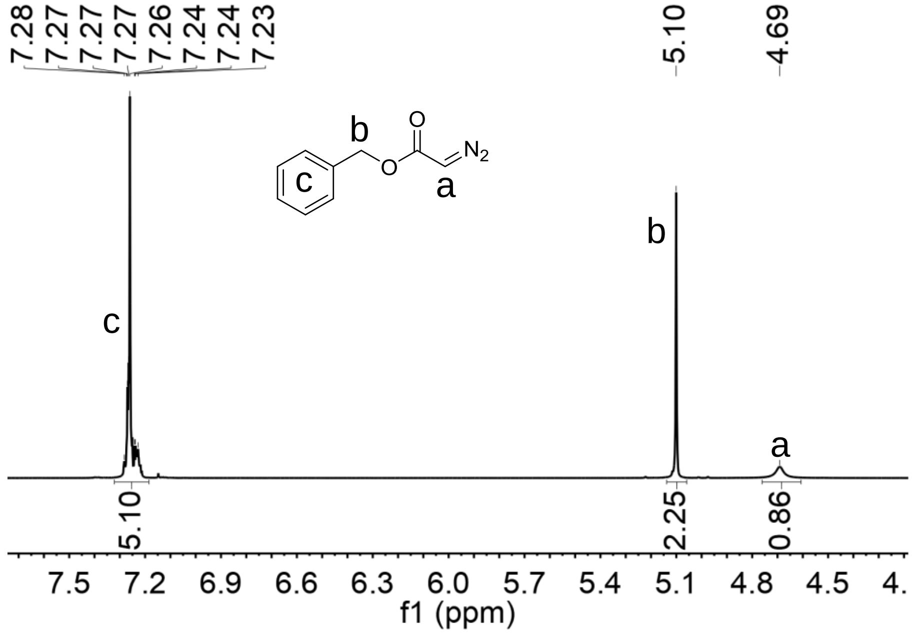

Supplementary Fig. 38 ^{1}H NMR (600 MHz) spectrum of **1a** recorded in $CDCl_3$ at room temperature.

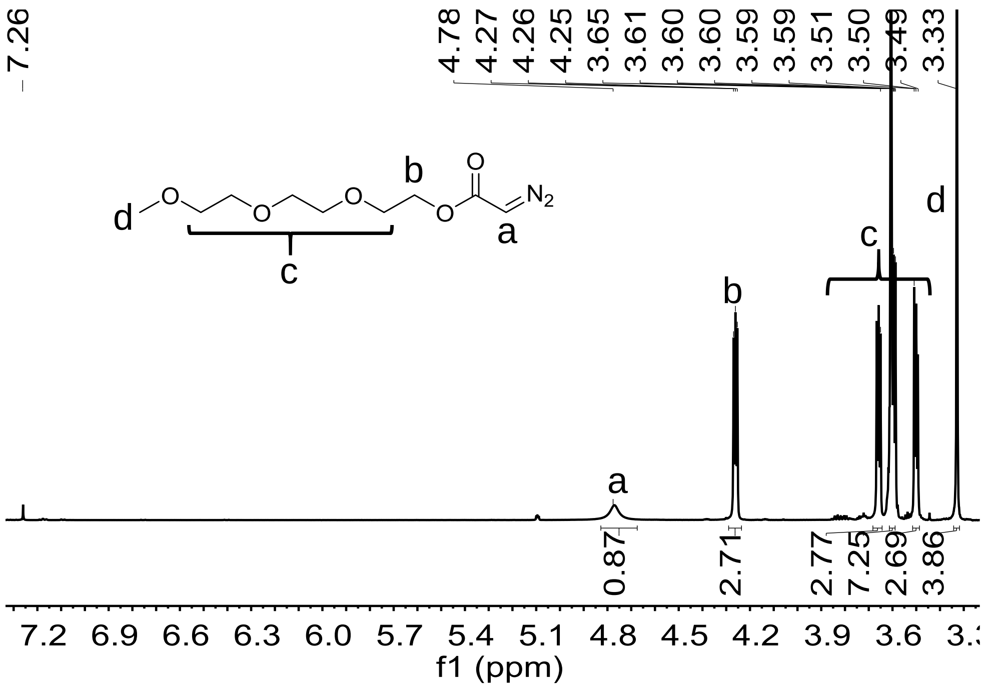

Supplementary Fig. 39 ^{1}H NMR (600 MHz) spectrum of **1b** recorded in $CDCl_3$ at room temperature.

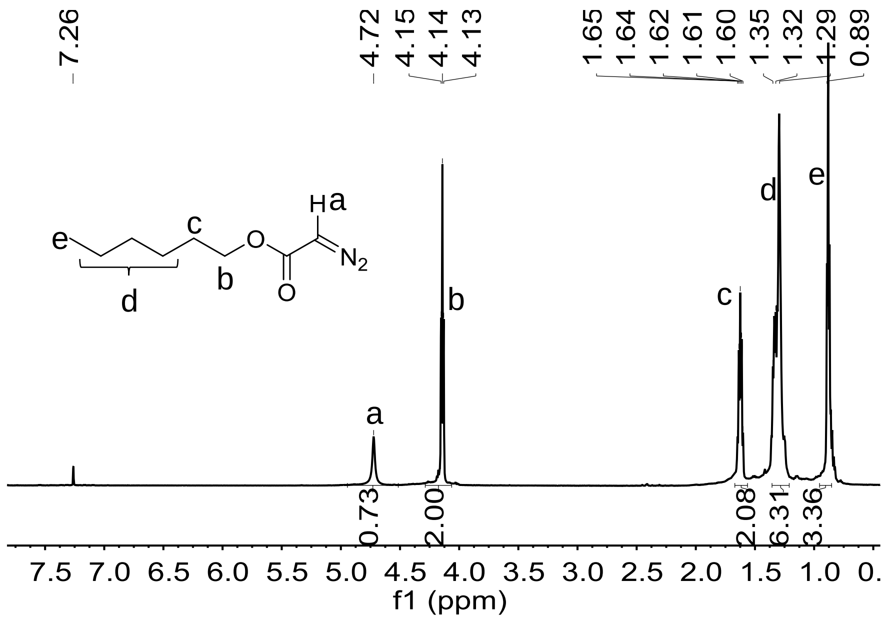

Supplementary Fig. 40 ^{1}H NMR (600 MHz) spectrum of **1d** recorded in $CDCl_3$ at room temperature.

Supplementary references

1. Chu, J.-H., Xu, X.-H., Kang, S.-M., Liu, N. & Wu, Z.-Q. Fast Living Polymerization and Helix-Sense-Selective Polymerization of Diazoacetates Using Air-Stable Palladium(II) Catalysts. *J. Am. Chem. Soc.* **140**, 17773–17781 (2018).
2. Yu, Z.-P., Ma, C.-H., Wang, Q., Liu, N., Yin, J. & Wu, Z.-Q. Polyallene-*block*-polythiophene-*block*-polyallene Copolymers: One-Pot Synthesis, Helical Assembly, and Multiresponsiveness. *Macromolecules* **49**, 1180–1190 (2016).
3. Zhu, Y.-Y., Yin, T.-T., Li, X.-L., Su, M., Xue, Y.-X., Yu, Z.-P., Liu, N., Yin, J. & Wu, Z.-Q. Synthesis and Chiroptical Properties of Helical Polyallenes Bearing Chiral Amide Pendants. *Macromolecules* **47**, 7021–7029 (2014).
4. Siriwardane, D. A., Kulikov, O., Reuther, J. F. & Novak, B. M. Rigid Helical Arm Stars through Living Nickel Polymerization of Carbodiimides. *Macromolecules* **50**, 832–840 (2017).
5. Yokoyama, A., Miyakoshi, R. & Yokozawa, T. Chain-Growth Polymerization for Poly(3-hexylthiophene) with a Defined Molecular Weight and a Low Polydispersity. *Macromolecules* **37**, 1169–1171 (2004).
6. Ye, S., Cheng, S., Pollit, A. A., Forbes, M. W. & Seferos, D. S. Isolation of Living Conjugated Polymer Chains. *J. Am. Chem. Soc*. **142**, 11244–11251 (2020).
7. Wu, Z.-Q., Chen, Y., Wang, Y., He, X.-Y., Ding, Y.-S. & Liu, N. One pot synthesis of poly(3-hexylthiophene)-*block*-poly(hexadecyloxyallene) by sequential monomer addition. *Chem. Commun*. **49**, 8069–8071 (2013).
